# Inclusion of the Phytoalexin *trans*-Resveratrol in Native Cyclodextrins: A Thermal, Spectroscopic, and X-Ray Structural Study

**DOI:** 10.3390/molecules25040998

**Published:** 2020-02-24

**Authors:** Laura Catenacci, Milena Sorrenti, Maria Cristina Bonferoni, Lee Hunt, Mino R. Caira

**Affiliations:** 1 Department of Drug Sciences, University of Pavia, viale Taramelli 12, 27100 Pavia, Italy; laura.catenacci@unipv.it (L.C.); mariacristina.bonferoni@unipv.it (M.C.B.); 2 Centre for Supramolecular Chemistry Research, Department of Chemistry, University of Cape Town, Rondebosch 7701, South Africa; trllee001@gmail.com

**Keywords:** cyclodextrin, *trans*-resveratrol, inclusion complexes, thermal analysis, X-ray structure

## Abstract

The aim of the study was to determine the feasibility of complexation between the antioxidant *trans*-resveratrol (RSV) and underivatized cyclodextrins (CDs) using a variety of preparative methods, including physical mixing, kneading, microwave irradiation, co-evaporation, and co-precipitation techniques. Products were characterized using differential scanning calorimetry (DSC), simultaneous thermogravimetric/DSC analysis (TGA/DSC), Fourier transform infrared (FT-IR) spectroscopy, and powder X-ray diffraction (PXRD). With α-CD and RSV, sample amorphization was revealed by PXRD and FT-IR, but no definitive inclusion complexation was evident. Similar results were obtained in attempts to complex RSV with β-CD. However, complex formation between γ-CD and RSV was evident from observation of an endo-/exothermic effect appearing in the DSC trace of the product from kneading and was further corroborated by FT-IR and PXRD methods. The latter technique indicated complexation unequivocally as the diffraction peak profile for the product matched that for known isostructural γ-CD complexes. Single crystal X-ray analysis followed, confirming the predicted complex between γ-CD and RSV. A combination of ^1^H NMR and TGA data yielded the complex formula (γ-CD)_3_·(RSV)_4_·(H_2_O)_62_. However, severe disorder of the RSV molecules prevented their modeling. In contrast, our previous studies of the inclusion of RSV in methylated CDs yielded crystals with only minor guest disorder.

## 1. Introduction

**Figure 1:**
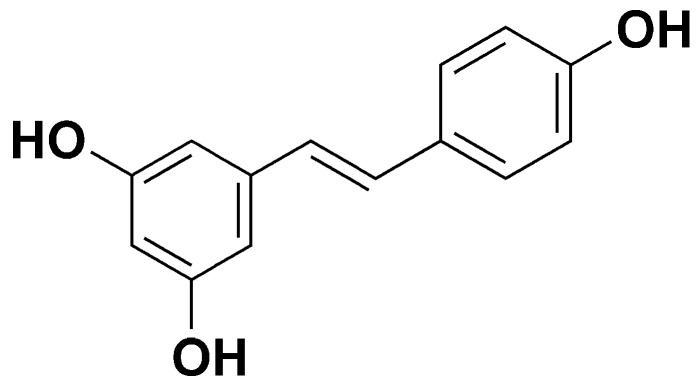
Chemical structure of *trans*-resveratrol.

*Trans*-resveratrol (*trans*-3,5,4′-trihydroxystilbene, RSV) (Figure 1) is a triphenolic phytoalexin with potent antioxidant activity and is found in a variety of plant species, such as grapevines, mulberries, peanuts, and the dried roots and stems of Japanese knotweed *(Polygonum cuspidatum)*. An increasing volume of research on RSV has revealed its diverse range of biomedical properties, which may indicate its use for the treatment of a variety of human diseases [1,2,3,4,5].

It has been claimed that RSV has an anti-inflammatory effect, improves heart health, prevents cancer, and protects against neurological diseases, among other benefits, such as anti-aging, anti-diabetic, anti-obesity, and skin photoprotection activity. Despite promising results in preclinical settings, the applicability of RSV to humans has met with only limited success, largely owing to its inefficient systemic delivery and consequently its low oral bioavailability due to adverse physicochemical properties, such as low stability, increased oxidation on heat and light exposure, and poor aqueous solubility [6].

Bioavailability enhancement can be achieved using a different solid form of a drug or nutraceutical, for example, an amorphous form in lieu of a crystalline form. In this context, the present study focused primarily on solid form screening, with the aim of isolating and characterizing possible new forms of RSV (unsolvated, solvated, hydrated, and amorphous materials). Different solid forms could display differences in their physicochemical properties, such as solubility and chemical stability, with varied impact on pharmaceutical and biopharmaceutical properties [7,8,9,10]. The solid state of RSV is an important aspect to consider during preformulation, formulation, and manufacturing processes, especially considering the possibility of the conversion of *trans*-RSV into *cis*-RSV, the latter being the biologically less active isomer [11]. Seven crystal structures of *trans*-RSV (not all unique) are reported in the Cambridge Structural Database (CSD [12]), version 5.40 updates, August 2019, with refcodes DALGON and DALGON01-DALGON06. These include structures determined from single crystals and powders. Further reference is made to these crystal forms below in the context of RSV polymorph identification using the PXRD method.

Considering that drug polymorphs can often be discovered simply by changing the solvent system, in the first phase of the present study, solid forms of RSV were prepared by recrystallization of the commercial material from hot saturated solutions in different solvents and by submitting the compound to typical processing steps in pharmaceutical manufacturing, namely kneading, microwave irradiation, and co-evaporation processes.

Furthermore, since the use of RSV in combination with native and modified cyclodextrins (CDs), used as additives and drug-complexing agents, could be very useful to overcome the limitations in the use of RSV, enhancing its bioavailability and reducing its perceived toxicity [13,14,15], their employment in this study was also considered relevant. CDs are cyclic oligosaccharides containing six (α-CD), seven (β-CD), eight (γ-CD), or more α-1,4-linked α-D-glucopyranose units, obtained from the enzymatic degradation of starch by *Bacillus macerans*. Native CDs and their derivatives, such as hydroxypropyl-β-CD (HPβCD), sulfobutyl ether β-CD (SBEβCD), and dimethyl β-CD (DMB), which possess higher aqueous solubility, are widely used in the pharmaceutical field, owing to their ability to solubilize and stabilize drug molecules [16,17,18,19]. While applications for drug delivery are the ultimate goals, their viability is entirely dependent on the feasibility of isolating well-defined inclusion complexes that can be incorporated into appropriate formulations (e.g., tablets, capsules).

Various methods are used to prepare CD–drug inclusion complexes. They include solution-phase techniques, such as co-precipitation and evaporation; the kneading method, which yields a paste, and mechanical grinding methods that produce a solid phase [20,21]. The mechanical activation of solid-state mixing or kneading can cause CD–drug interaction or complexation, resulting in the modification of physicochemical properties, such as the dissolution rate and bioavailability of the encapsulated molecule.

*Trans*-RSV is reported to form inclusion complexes with native and modified CDs but the literature on this subject is confusing; complexation has been studied primarily in solution [14,22], and as far as the solid state is concerned, the characterization is incomplete [23]. In a previous paper, we reported the solid-state characterization by thermal analysis of inclusion complexes of *trans*-RSV in methylated CDs, specifically permethylated α-CD, permethylated β-CD and 2,6-dimethylated β-CD. Isolation of the corresponding crystalline 1:1 inclusion complexes enabled their full structure determination by X-ray analysis for the first time, revealing a variety of guest inclusion modes and unique supramolecular crystal packing motifs [24].

In the study reported here, the products of interaction between RSV and native CDs were prepared via physical mixing, kneading, co-precipitation, or co-crystallization from different solutions, co-evaporation using a rotary evaporator, or exposure to microwave irradiation. The effects of the various preparative methods on the inclusion complexation were investigated by means of differential scanning calorimetry (DSC), simultaneous thermogravimetric analysis (TGA/DSC) with support from Fourier transform infrared (FT-IR) spectroscopy, and powder X-ray diffraction (PXRD). An important goal of the present study was the isolation and detailed characterization of the γ-CD inclusion complex of RSV, owing to the pharmaceutical advantages of the host γ-CD, namely its high aqueous solubility relative to the other native CDs as well as its lower toxicity [20].

## 2. Results

### 2.1. Thermal, PXRD, and FT-IR Characterization of Prepared Solid Phases

**Figure 2:**
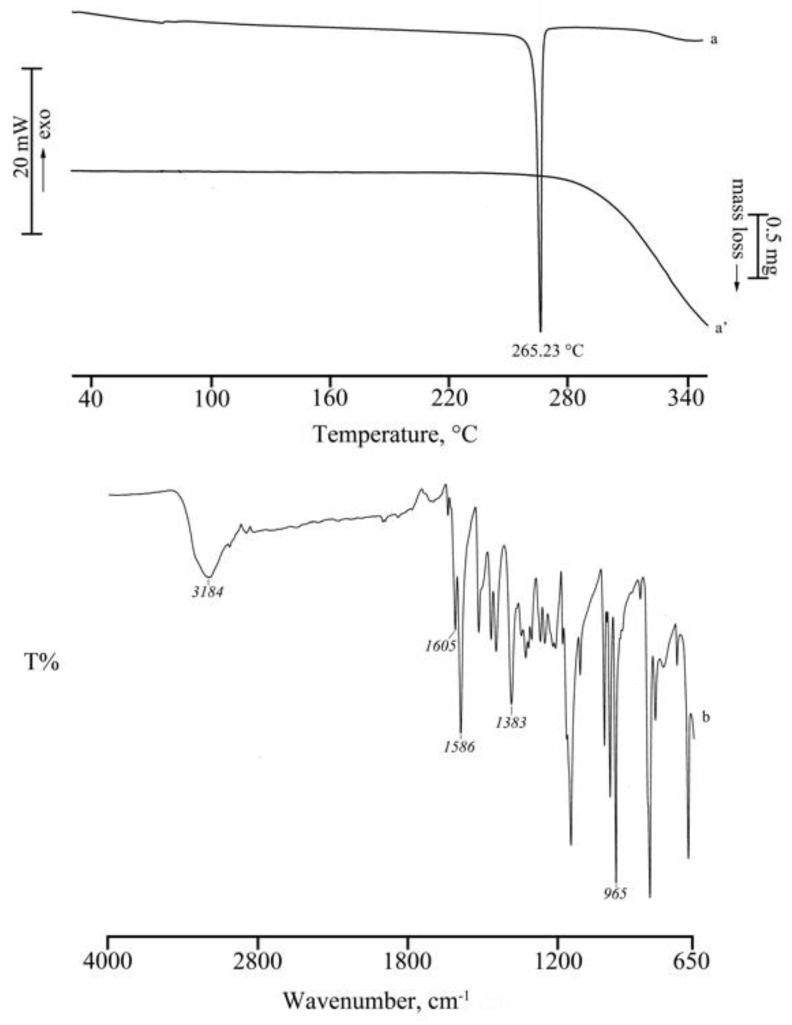
DSC and TGA curves (a, a’) and FT-IR spectrum (b) of commercial RSV.

In our study of the inclusion of RSV in methylated CDs [24], we presented comprehensive quantitative thermal and FT-IR data for the commercially available sample of RSV used in the present study. Figure 2 shows the respective TGA and DSC traces (curves a’, a) as well as the FT-IR spectrum (b) of RSV. These serve as reliable references for identifying the pure bioactive compound.

**Figure 3:**
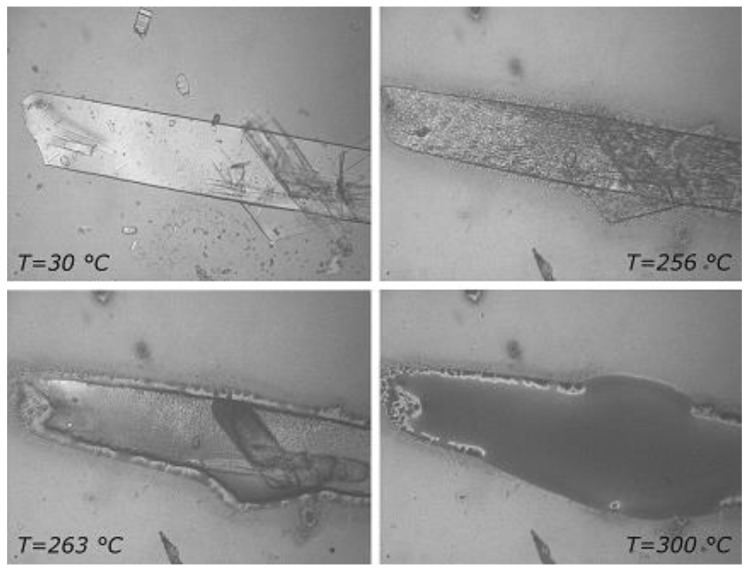
HSM images of RSV recrystallized from methanol–water solution, at the various temperatures reported on the images.

Table S1 (Supplementary Material, File 1) lists pertinent thermal parameters of the solid phases obtained by recrystallization of commercial RSV from a variety of pure solvents and various aqueous solutions. These data confirm that all the recrystallized samples of RSV isolated correspond to the same polymorphic form, which we also determined corresponds to the commercial material employed. However, the data in Table S1 also indicate different degrees of crystallinity for these samples, as is evident from variation in their enthalpies of fusion. Furthermore, some extent of overlap of the decomposition and melting processes also has to be taken into consideration, as it contributes to inaccuracy in the evaluation of the enthalpies. Figure 3 shows, as an example, HSM (hot stage microscopy) images of the sample obtained by recrystallization of RSV from MeOH/H_2_O (7:3 *v*/*v*). The crystals displayed blade-like morphology at room temperature and on heating shrank slightly but retained their original shape until they melted at approximately 260 °C, prior to decomposing.

We reported previously that RSV itself does not undergo any significant changes in solid-phase characteristics and/or physical stability upon treatment with procedures, such as kneading (KN), microwave irradiation (MW), and co-precipitation (CP) [24]. This was established prior to undertaking the screening experiments with binary mixtures of RSV and various native CDs in attempts to isolate new solid forms of the bioactive compound using the above preparative conditions. The results of these experiments are presented below.

#### 2.1.1. RSV + α-CD

**Figure 4:**
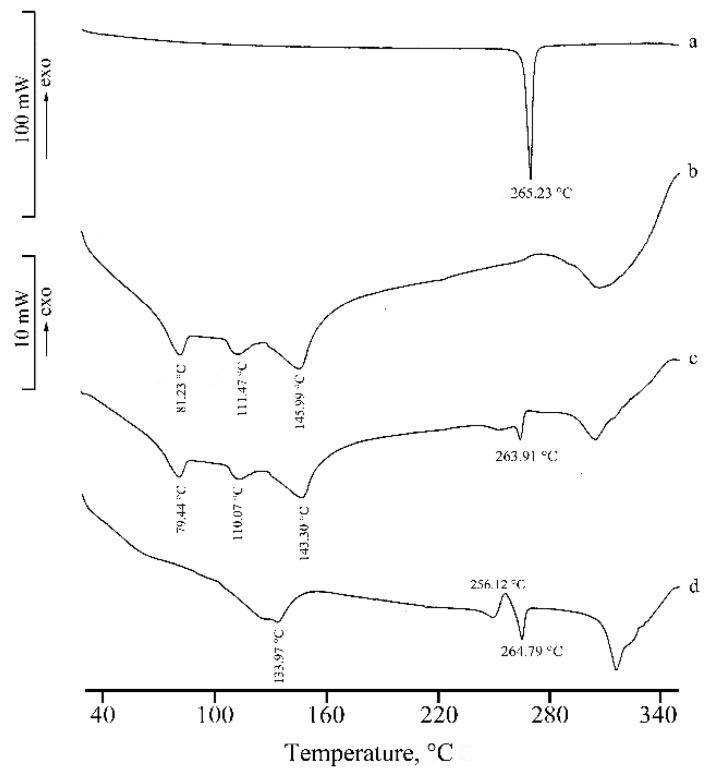
DSC curves of RSV (**a**), α-CD (**b**), their PM (**c**), and KN product (**d**).

Figure 4 shows the DSC curves of RSV (a), α-CD (b), their physical mixture (PM) (c), and the product from KN (d). In the DSC curve of α-CD, three distinct effects are present in the temperature range 30–160 °C, attributed to sample dehydration with TGA overall mass loss of 10.3(4)% (curve not shown), in agreement with the theoretical value (10.0%), followed by melting with concomitant decomposition at around 270 °C. The presence of the drug melting endotherm is evident in both the DSC curve of the PM and in the trace following the kneading treatment to which the binary mixture was submitted. This effect was accompanied by an endo-exothermic effect at 249 and 256 °C, respectively, probably due to partial amorphization of the sample.

**Figure 5:**
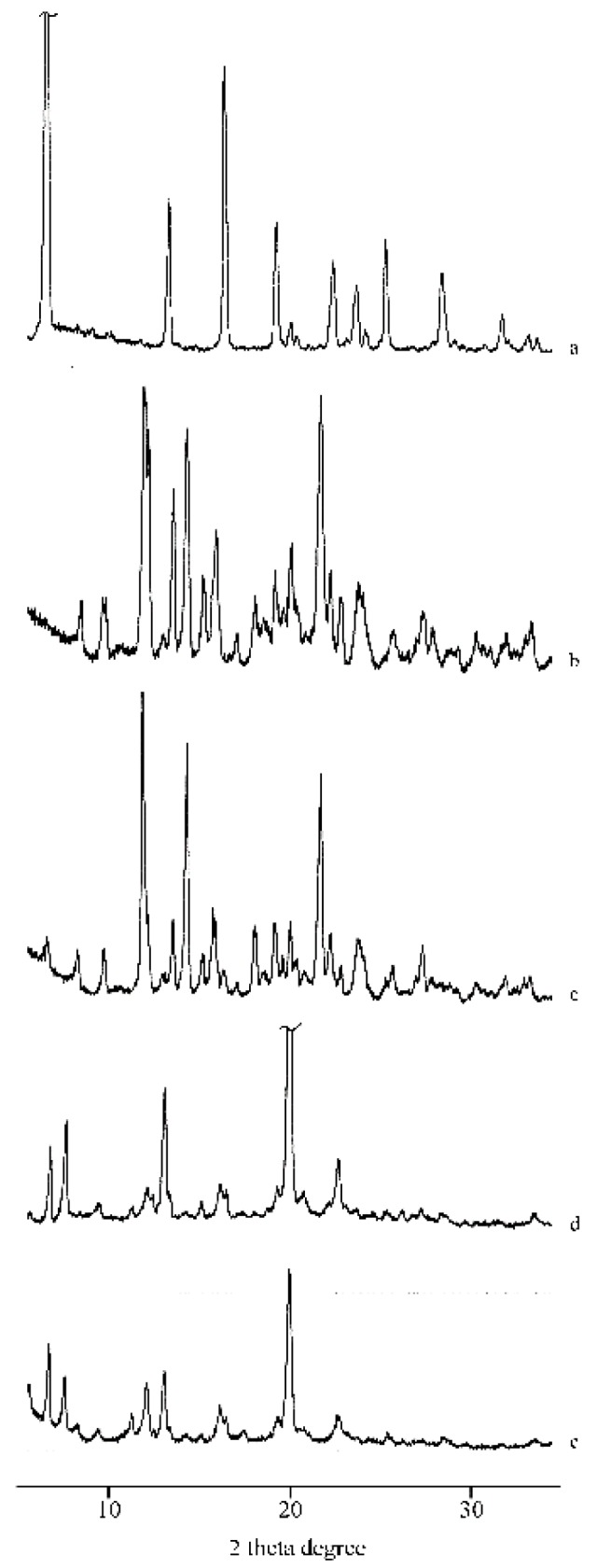
PXRD patterns of RSV (**a**), α-CD (**b**), their PM (**c**), MW (**d**), and CP (**e**) products.

The PXRD patterns indicated this amorphization after MW and CP treatments (Figure 5, patterns d and e), caused by sample preparation, compared to their PM (pattern c) where the diffraction peaks characteristic of RSV and α-CD are present unmodified.

**Figure 6:**
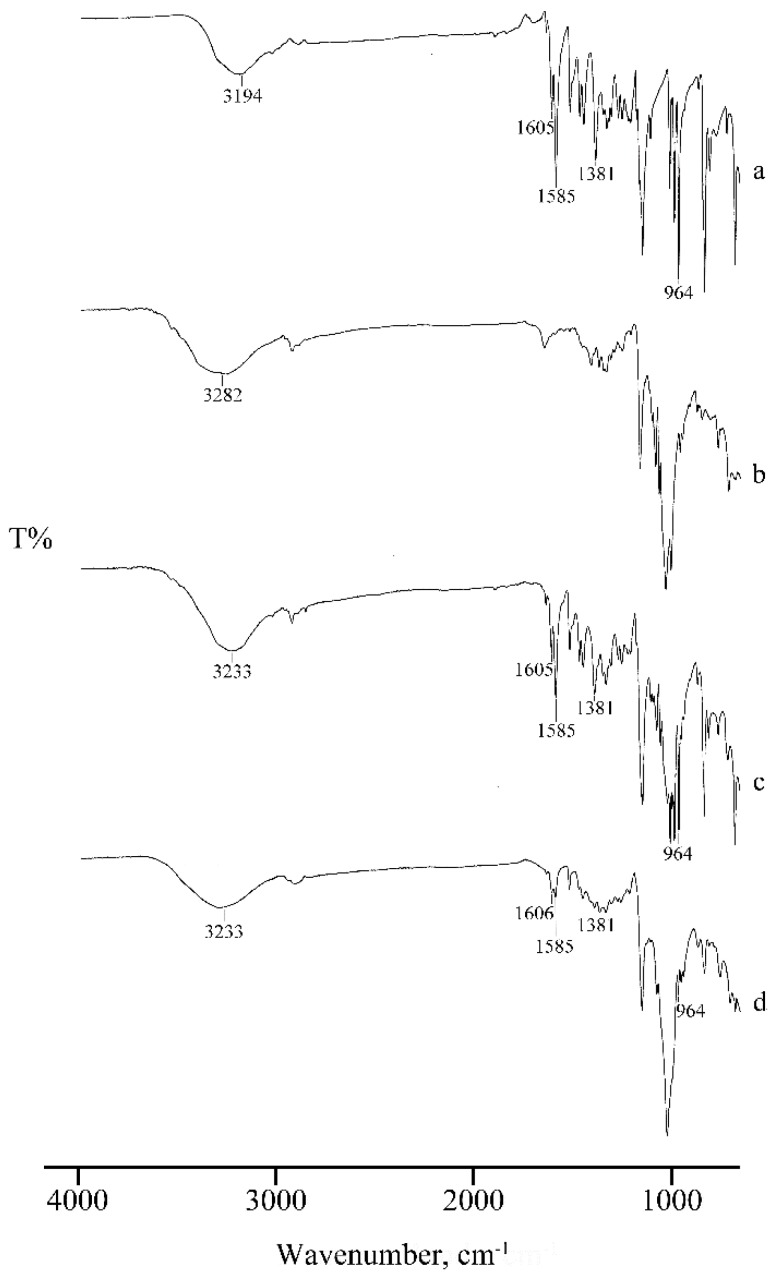
FT-IR spectra of RSV (**a**), α-CD (**b**), their PM (**c**), and MW product (**d**).

This effect is also confirmed by the broadening of the absorption bands in the FT-IR spectrum of the MW product (Figure 6, spectrum d), where the characteristic bands of RSV are present at unchanged wavenumbers but with lower intensity.

As noted above, the PXRD method was useful in demonstrating amorphization of RSV samples. However, it was also useful for verifying the integrity of the PXRD pattern of the RSV sample shown as a reference in Figure 5a. To establish this, the angular positions of major peaks in its PXRD pattern were compared with those that we computed for all known RSV forms (CSD refcodes DALGON [25], DALGON01-DALGON03 [26], DALGON04 [27], DALGON05 [28], and DALGON06 [29]). Good correlation was found with the PXRD patterns of DALGON, DALGON04 (revised structure of DALGON), DALGON05, and DALGON06. Ignoring H-bonding and disorder issues, these four structures are essentially the same, having the common space group *P*2_1_/c and similar unit cell dimensions, except that the *a*-axis length in DALGON is approximately doubled in the other three structures. The latter phenomenon is common, revealing superlattice reflections (as for DALGON04 [28]), but changing the overall crystal structure in only a subtle manner, and hence producing crystalline phases with very similar PXRD patterns.

#### 2.1.2. RSV + β-CD

**Figure 7:**
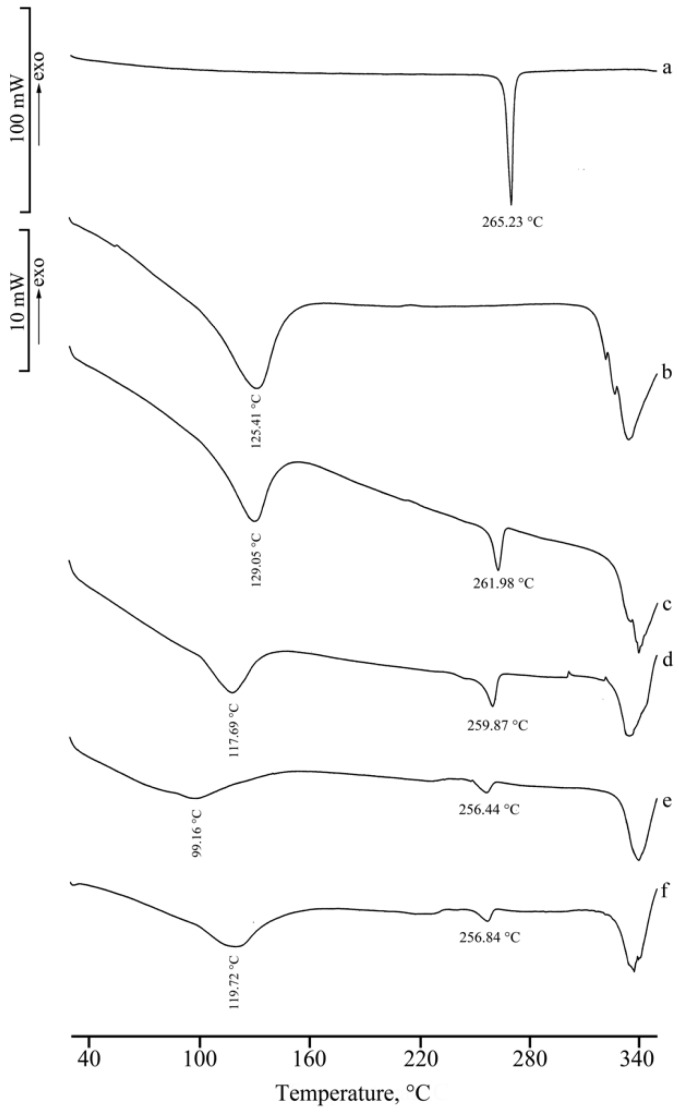
DSC curves of RSV (**a**), β-CD (**b**), their PM (**c**), and KN (**d**), MW (**e**), and CP (**f**) products.

In the binary system with the higher homologue β-CD, the presence of the endothermic effect due to the melting of RSV in the DSC profiles of the PM and all the treated products indicate the absence of interaction between RSV and the host carrier (Figure 7). The fusion enthalpy in all the treated samples was reduced by 50% (ΔH_m_ = 124(2) J·g^−1^) relative to that of pure RSV, as a consequence of the amorphization of the system; this is confirmed also by the broadening of the diffraction peaks in the PXRD patterns recorded for the systems (patterns not shown).

#### 2.1.3. RSV + γ -CD

**Figure 8:**
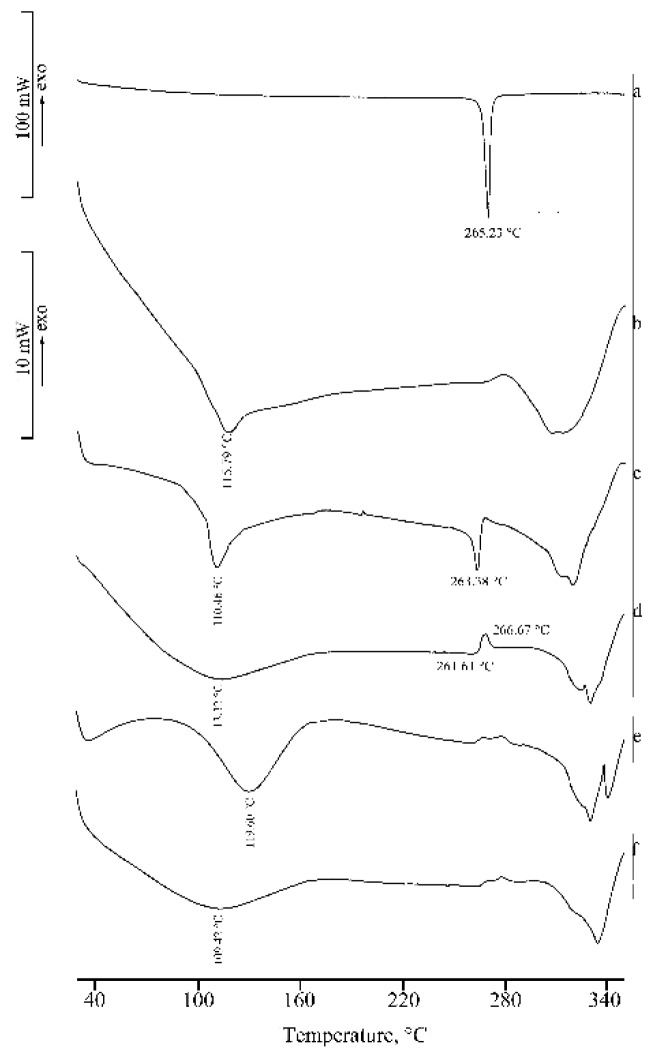
DSC curves of RSV (**a**), γ-CD (**b**), their PM (**c**), and products from KN (**d**), MW (**e**), and CP (**f**) treatments.

Figure 8 presents the DSC curves of RSV (a), γ-CD (b), their PM (c), and the latter after treatments by KN (d), MW (e), and CP (f), respectively. γ-CD is a crystalline sample characterized by a DSC curve showing a broad dehydration endotherm (T_peak_ = 115(3) °C), which corresponds to a TGA mass loss of 8.3(6)% (curve not shown) and melting with decomposition at ~300 °C. The melting endotherm of RSV is still present in the DSC profile of the PM, although with melting enthalpy value much lower than that of the pure material (ΔH_m_ = 30(2) J·g^−1^) because of a solid-state interaction between RSV and the CD. However, the RSV melting endotherm is not evident in the treated samples and instead an endo-/exothermic effect at ~260 °C appears in their DSC traces, being more pronounced in the KN product (d) and attributed to inclusion complex formation between RSV and the carrier.

**Figure 9:**
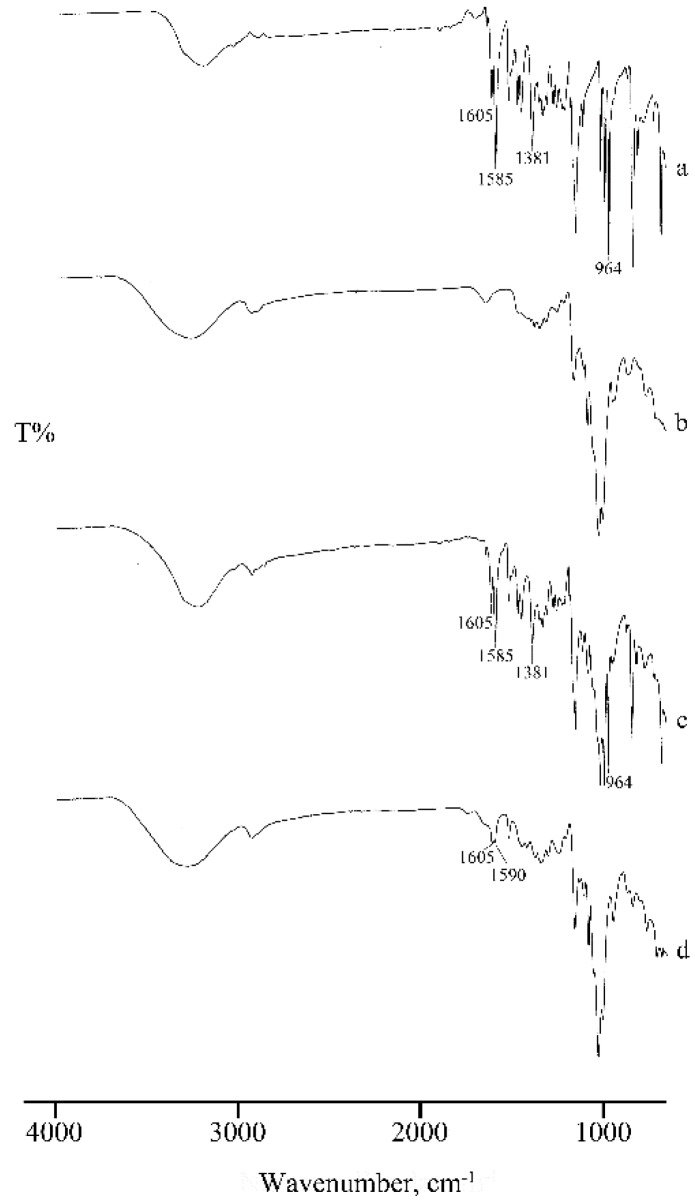
FT-IR spectra of RSV (**a**), γ-CD (**b**), their PM (**c**), and KN product (**d**).

In the FT-IR spectra (Figure 9) recorded on the same samples, the typical absorption bands of RSV are present in the spectrum of the PM (c) but absent, or shifted to slightly higher wavenumbers, in the KN product (d), suggesting RSV inclusion in the cavity of γ-CD, supporting the interpretation above based on the thermal data.

**Figure 10:**
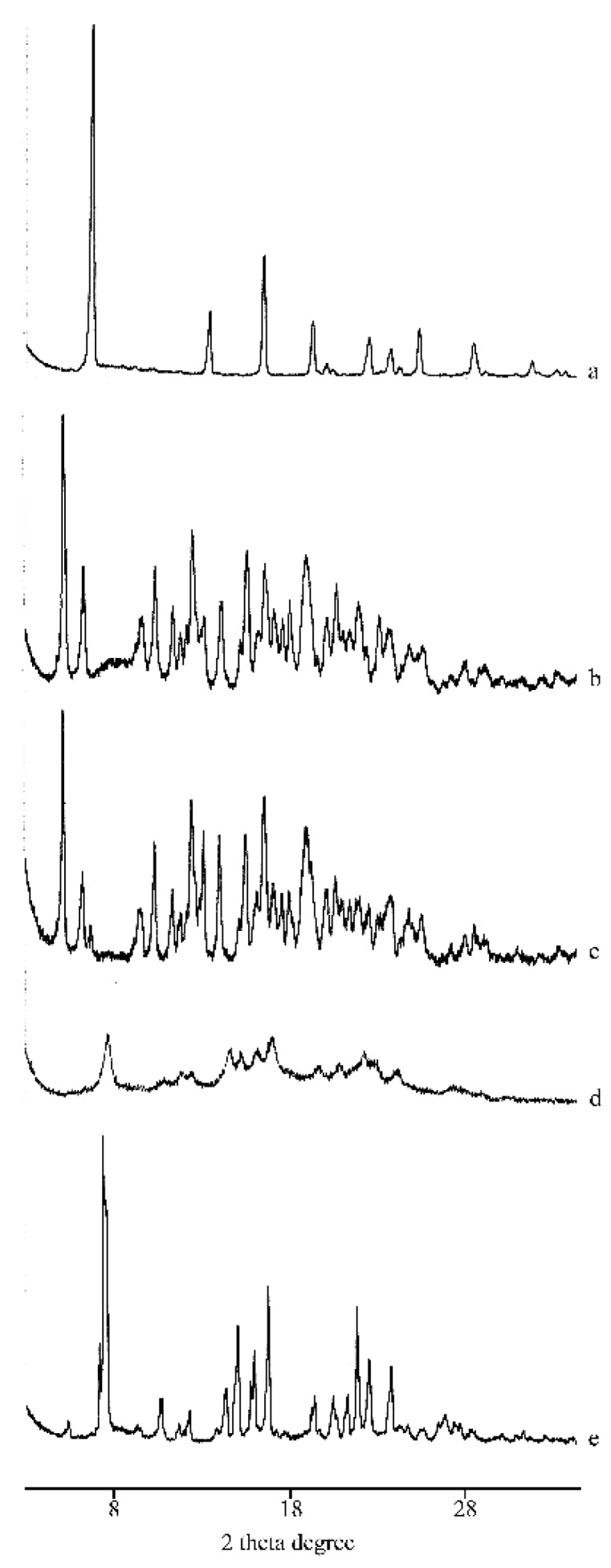
PXRD patterns of RSV (**a**), γ-CD (**b**), their PM (**c**), KN(**d**), and the CCP (**e**) products.

Figure 10 shows the PXRD patterns recorded for the bulk materials (patterns a and b) compared with their PM (pattern c) or after KN treatment (pattern d). The presence of a new series of peaks in the pattern of the KN product confirmed the interaction between RSV and γ-CD revealed by thermal analysis and FT-IR spectroscopy. The pattern (d) represents the same phase as that of pattern (e), the latter being a sample of higher crystallinity obtained by recrystallizing the PM from EtOH/H_2_O (1:1 *v*/*v*), the resulting product being labelled CCP (for ‘co-crystallization product’). As far as the feasibility of complexation is concerned, more definitive conclusions can be drawn in this instance because the detailed profile of the peaks in pattern (e) corresponds to a common PXRD pattern displayed by the well-known isostructural series of tetragonal γ-CD inclusion complexes containing a variety of guest molecules [30]. This correspondence thus proves that the phase depicted in pattern (e) is a genuine γ-CD inclusion complex crystallizing in the tetragonal space group P42_1_2, for which the unit cell parameters should have values a ≈ 23.8 Å, c ≈ 23.0 Å, by analogy with the data for the isostructural series. These conclusions were subsequently confirmed by single crystal X-ray analysis of the γ-CD·RSV complex (Section 2.2).

**Figure 11:**
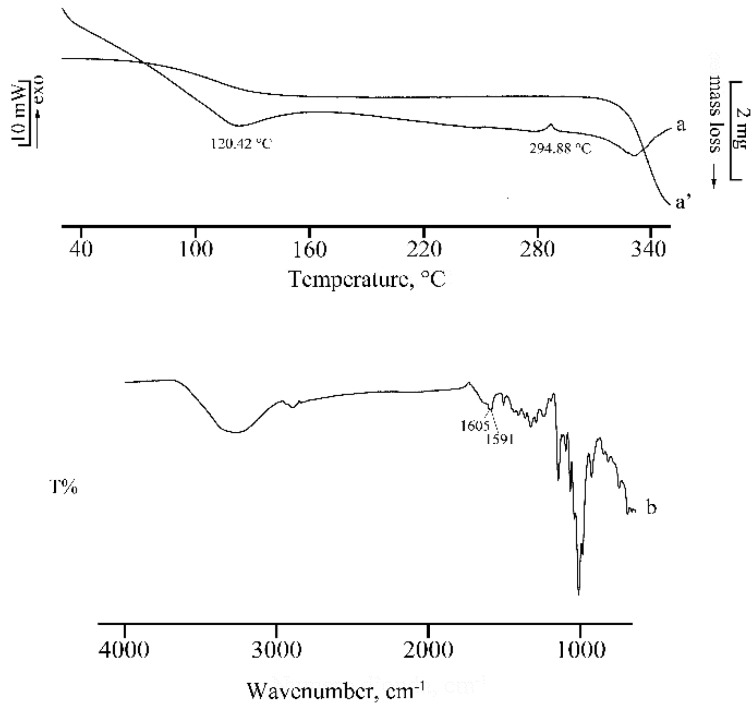
DSC and TGA curves (**a**, **a’**) for the γ-CD·RSV complex and its FT-IR spectrum (**b**).

Figure 11 reports the thermal profiles (curves a and a′) and the FT-IR spectrum (b) of the product obtained by recrystallization of the PM from the hydroalcoholic solution. The TGA mass loss, corresponding to desolvation, amounted to 17.4(4)% and was recorded over a wide temperature range between 80 and 170 °C. Desolvation is followed by complex decomposition at 295 °C. The DSC curve showed the existence of a new solid phase due to the complexation effect of γ-CD, as confirmed also by its FT-IR spectrum, which was superimposable on the spectrum already reported for the KN product (see Figure 9).

**Figure 12:**
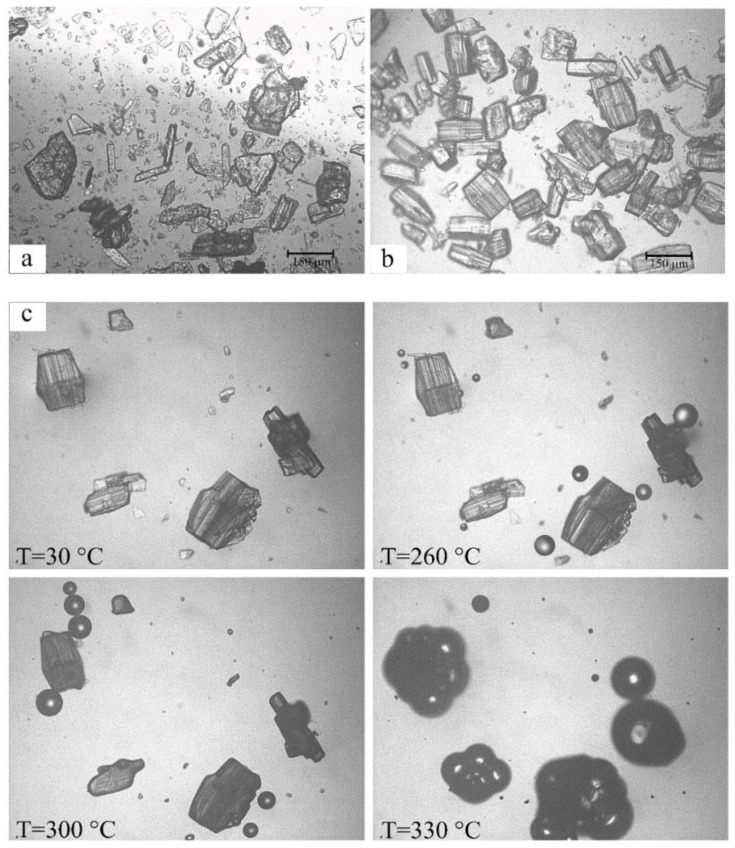
Photomicrographs of the RSV - γ-CD PM (**a**) and the CCP sample (**b**) at room temperature, and HSM micrographs of CCP at the various temperatures reported on the images (**c**).

In Figure 12, the photomicrograph recorded for the PM (a) shows two crystal phases, namely those of RSV and γ-CD, while the CCP product (b) shows the typical columnar and bipyramidal crystal morphologies displayed by tetragonal γ-CD inclusion complexes. The HSM images for the sample (c) confirmed the isolation of a new phase. During heating, the crystals lost their opacity and at ~160 °C, in accord with the sample desolvation recorded on TGA analysis, loss of the solvents of crystallization (water, ethanol) was observed as bubbles escaping into the silicone oil in which the sample was immersed. Decomposition began at 300 °C and the complex decomposed completely at 330 °C without any clear melting point, as is typical for γ-CD complexes.

### 2.2. X-Ray Structural Analysis of the γ-CD·RSV Complex

Crystals suitable for X-ray analysis were prepared using the co-precipitation method (details are provided in Section 4.3). The host-guest stoichiometry of the γ-CD·RSV complex was determined as 3:4 by ^1^H NMR spectroscopy of pure complex crystals dissolved in DMSO-d_6_ (Figure S1 and Table S2, Supplementary Material, File 1). From the TGA trace for these crystals (not shown), a mass loss percentage of 18.9(2)% was recorded, allowing determination of the composition of the ternary complex as (γ-CD)_3_·(RSV)_4_·(H_2_O)_62_. The single crystal X-ray analysis yielded the following results:

Crystal data for (γ-CD)_3_·(RSV)_4_·(H_2_O)_62_ (M = 5921.27 g/mol): Tetragonal, space group P42_1_2 (no. 90), a = 23.7589(12) Å, c = 22.9285(12) Å, V = 12942.8(15) Å^3^, Z = 2, T = 173(2)K, μ(MoKα) = 0.137 mm^−1^, D_calc_ = 1.519 g/cm^3^, 64155 reflections measured (2.5° ≤ 2Θ ≤ 47.8°), 9995 unique (R_int_ = 0.0907), which were used in all calculations. The final R_1_ was 0.1004 (I > 2σ(I)) and wR_2_ was 0.2963 (all data).

**Figure 13:**
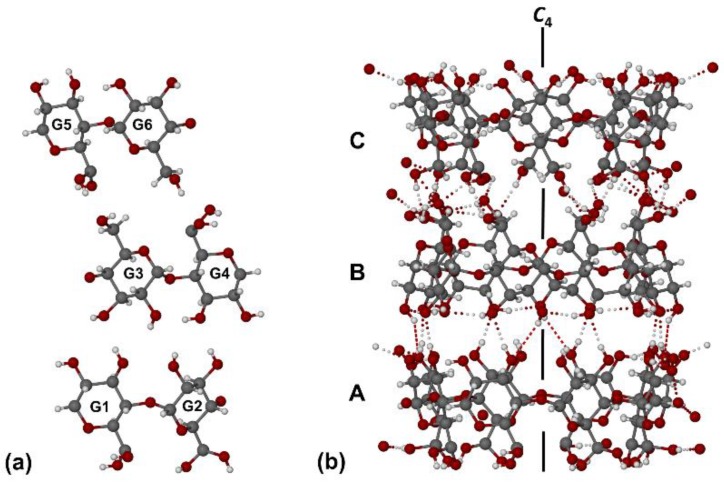
The three distinct pairs of glucopyranose units in the asymmetric unit of the (γ-CD)_3_·(RSV)_4_·(H_2_O)_62_ complex (**a**) and the resulting stacking of the three unique γ-CD molecules parallel to the crystal c-axis (**b**). Intramolecular and intermolecular O-H···O hydrogen bonds are indicated by dotted lines.

Inclusion complexes with the space group and unit cell dimensions above are known to crystallize with three crystallographically independent γ-CD molecules in the unit cell, each of these macrocycles being generated by a symmetry operation from two distinct linked glucopyranose residues (Figure 13a). The three unique γ-CD molecules in the complex with RSV are thus located on a fourfold rotation axis (C_4_), which is parallel to the crystal c-axis (Figure 13b). Each γ-CD molecule contains glucopyranose units in the ^4^C_1_ chair conformation and has the well-known overall shape of a truncated cone, with the wider secondary rim bearing 16 hydroxyl groups and the narrower primary rim bearing 8 hydroxyl groups. The sequence of molecules (ABC...) is repeated indefinitely along the z-direction, giving rise to three distinct types of interfacial contact, namely secondary rim···secondary rim (A···B), primary rim···primary rim (B···C), and secondary rim···primary rim (C···A´, where A´ represents molecule A translated by one repeat unit along the z-direction).

**Figure 14:**
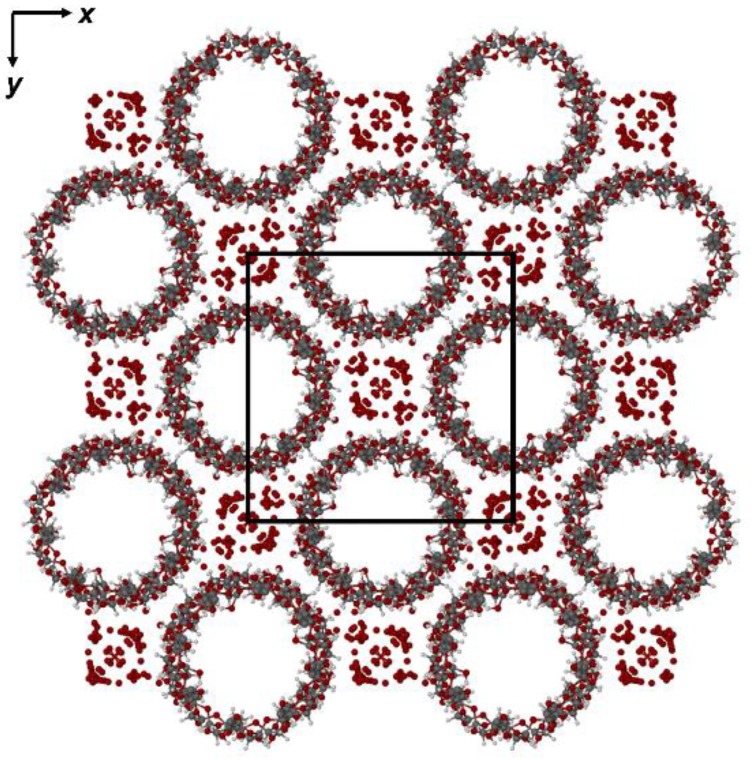
The [001] projection of the structure of the (γ-CD)_3_·(RSV)_4_·(H_2_O)_62_ complex.

A common drawback in the X-ray structural elucidation of γ-CD inclusion complexes crystallizing in the space group P42_1_2 is that the guest molecules are generally disordered since they are located within infinite channels of fourfold rotational symmetry generated by overlap of the central cavities of the stacked macrocyclic molecules. Exceptions to this general situation occur when the guest molecule itself possesses fourfold symmetry (e.g., 12-crown-4), resulting in an ordered crystal structure [31], this case being shown in Figure S2 in the Supplementary Material. In the case of the RSV complex presented here, following structural refinement of the host and water molecules, the highest difference electron density (Δρ) peak within the channel was only 0.75 eÅ^−3^ (about twice the value for an average hydrogen atom in a small-molecule structure) and no meaningful candidate guest peak connectivity was evident. This is typical of complexes in this isostructural series and a value of ~0.100 for the R_1_ factor (as recorded above for the RSV complex) is likewise typical, its relatively low value indicating that the host assembly is structurally very well-defined while the guest molecules contribute little to the overall X-ray scattering from the crystal due to their severe spatial disorder. Of the estimated ~21 water oxygen atoms per γ-CD molecule expected from the measured TGA mass loss for dehydration of the RSV complex, approximately 15 could be accounted for in the structural analysis and were added to the model. The majority of these also displayed partial occupancy. Figure 14 shows the projection of the crystal structure down the crystal c-axis. The disordered RSV molecules are present within the apparently ‘empty’ channels. Clusters of water oxygen atoms (small red spheres) occupy the interstitial sites. Water molecules engage in extensive hydrogen bonding with each other and with the infinite columns of γ-CD molecules, stabilizing the crystal structure.

As a check on the correctness of the X-ray model, the simulated PXRD pattern was calculated [32] using the space group data, unit cell dimensions, and refined atomic positions and thermal displacement factors. This pattern and the experimental PXRD pattern shown in Figure 10e appear as Figure S3 in the Supplementary Material, File 1. There is a clear 1:1 peak correspondence on comparing the simulated and experimental patterns. Intensity differences for the peaks at low angles are attributed to preferred orientation effects in the sample.

Following refinement of the above model involving discrete water molecules, the latter were removed and the SQUEEZE [33] procedure implemented in the program PLATON [34] was employed to refine the structure with the contributions from the water and guest RSV molecules removed from the diffracted intensities. As expected, this re-refinement resulted in significantly improved values of the final parameters, namely (R_1_ (0.0732), wR2 (0.2089) (I > 2σ(I)), S = 1.056 and Δρ limits 0.36, −0.32 eÅ^−3^. In addition, and more importantly, the value of the electron count per unit cell determined from the total (Fo-Fc) map following refinement based on the SQUEEZE approach (2 485), more than adequately confirmed the value predicted for the RSV and H_2_O content by the analytically derived stoichiometric formula (γ-CD)_3_·(RSV)_4_·(H_2_O)_62_ and Z = 2, namely 2200. The larger value of 2485 electrons per unit cell would account for 8 RSV molecules (960 e^−^) plus 152.5 water molecules (1525 e^−^) per unit cell. In terms of structural modelling, these data suggest that the total electron density due to water would account for ~19 H_2_O per crystal asymmetric unit as opposed to the 15.5 H_2_O located in the electron density map and included in the ‘discrete’ model. However, we place more reliance on the ^1^H NMR and TGA data for accurate estimation of the RSV and water contents, respectively.

## 3. Discussion

The present investigation focused on the feasibility of including the antioxidant *trans*-resveratrol (RSV) in the native cyclodextrins α-, β-, and γ-CD, the results indicating little or no evidence of the affinity of RSV to complex with either α- or β-CD under the conditions described and with the variety of preparative techniques employed. However, a definitive indication of complexation between RSV and γ-CD was first evident from thermal analysis and subsequently corroborated by FT-IR and ^1^H NMR spectroscopy, as well as PXRD and single crystal X-ray diffraction methods. Fortuitously, of the three native CDs investigated here for complexation with RSV, γ-CD possesses the highest aqueous solubility and lowest toxicity [35], which are advantageous from the pharmaceutical point of view. 

Predicting whether a given guest molecule will be included within the cavity of a specific CD is challenging due to the current imperfect level of understanding of the complex nature of the molecular interactions involved in the inclusion process, and although computational methods may suggest possible modes of guest inclusion for a given host–guest pair, such modes may be unrealistic if the medium (e.g., liquid/other phase) is not take into account explicitly in the calculations. Our previous study of the reactions between RSV and a series of methylated CDs using analogous preparative methods to those described here resulted in the isolation of its inclusion complexes with permethylated α-CD (TMA), 2,6-dimethyl β-CD (DMB) and permethylated β-CD (TMB) [24]. X-ray structures of these phases revealed insertion of the sterically less bulky 4-hydroxyphenyl ring of RSV into the cavities of the respective CDs from their secondary sides and in two cases (with TMA and DMB), the phenolic group of RSV is linked to the CD host via water-mediated hydrogen bonding of the type RSV(4-OH)···O(water)···O6(primary methoxy). Instead, in the TMB complex, there is a direct H-bond RSV(4-OH)···O6(primary methoxy) linking host and guest molecules. These results illustrate the unpredictability of the precise mode of inclusion of RSV in a given CD host molecule. The level of structural detail considered above is of course absent in the case of the (γ-CD)_3_·(RSV)_4_·(H_2_O)_62_ complex due to severe guest disorder. Nevertheless, the first step, namely isolating the complex between γ-CD and RSV and determining its composition, has been successful and is a prerequisite for its possible eventual formulation for drug delivery. Guest disorder in the crystals of (γ-CD)_3_·(RSV)_4_·(H_2_O)_62_ therefore does not negate the potential utility of the characterized complex in future studies aimed at determining whether the encapsulation of RSV in γ-CD has any effect on the bioavailability of the antioxidant in animal models.

## 4. Materials and Methods

### 4.1. Materials

The *trans*-resveratrol (RSV) sample used for most of the experiments was generously donated by Denk Feinchemie GmbH (München, Germany). For single crystal preparation, RSV purchased from Sigma Aldrich (South Africa) was employed. The native CDs were supplied by Wacker Chemie Italia Srl (Milan, Italy) and Cyclolab (Budapest, Hungary). All other materials and solvents used were of analytical reagent grade.

### 4.2. Recrystallization of RSV from Various Solvents

Samples were prepared by recrystallization of commercial RSV from different solutions; the solvents and the volumes used are indicated in Table S1 (Supplementary Material, File 1). The solutions were filtered and allowed to recrystallize by spontaneous cooling at ambient temperature. The resulting crystals were filtered and dried in a desiccator containing P_2_O_5_ under vacuum. The samples were subsequently protected from light to avoid their decomposition.

### 4.3. Preparation of the Binary Systems

The preparative methodology, presented in extenso in a previous paper [24], included isolation of physical mixtures (PMs), and the preparation of samples by kneading (KN), co-evaporation (CP), and treatment with microwave irradiation (MW), all involving equimolar samples of RSV and the relevant CD. For all of the above methods, the final procedural step was sieving the product through a 250-μm sieve. In addition, a common solvent medium, namely ethanol/water 4:1 (*v*/*v*), was employed in the KN, CP, and MW preparations.

Physical mixtures (PMs) of RSV and various CDs were prepared by manual trituration of samples comprising equimolar amounts of each component using a mortar and pestle. 

Products obtained by the kneading (KN) method were prepared by wetting each PM in the solvent medium and manually grinding the sample with a pestle. This was followed by drying each product to constant mass at 70 °C in an oven. For each product, the full procedure was performed in triplicate. 

To obtained co-evaporated (CP) products, each PM was dissolved in a minimum volume of the solvent medium. The latter was removed using a rotavapor (Büchi, Milan, Italy) under reduced pressure at 80 °C, yielding a residue that was triturated gently (mortar and pestle).

Microwave irradiation products (MP) were prepared by dissolving each PM in the minimum amount of the common solvent medium in a glass container and then irradiating the sample (425 W) with a Pabish CM-Aquatronic instrument (W. Pabish, Milan, Italy) until the solvent was removed. The dried residue was gently triturated (mortar and pestle).

Crystals of the γ-CD inclusion complex of RSV suitable for single-crystal X-ray diffraction were obtained by the co-precipitation procedure. This involved pre-dissolving 20 mg (0.088 mmol) RSV in 2 mL of a 1:1 *v*/*v* water-ethanol solution. This solution was added to a hot (50–60 °C) solution of γ-CD prepared by dissolving 124 mg (0.088 mmol) of the host in 2 mL of water at elevated temperature with stirring. The resulting solution was filtered into a clean vial and allowed to cool spontaneously at ambient temperature. Colorless crystals with tetragonal morphology were harvested after one week.

### 4.4. Thermal and Spectroscopic Analytical Methods

Temperature and enthalpy values were measured using a Mettler STAR^e^ system (Mettler Toledo, Novate Milanese, MI, Italy) fitted with a DSC821^e^ Module. Samples in the range 2–4 mg were weighed on a Mettler M3 Microbalance and placed in sealed Al pans with pierced lids [N_2_-atmosphere (flow rate 50 mL min^−1^), temperature range 30–350 °C, heating rate β = 10 K min^−1^]. Indium was used for instrument calibration and measurements were recorded in triplicate. For CCP and crystalline products, traces were recorded on a DSC-Q200 differential scanning calorimeter (TA Instruments, New Castle, DE, USA) with samples in closed Al pans heated at 10 K min^-1^ and dry N_2_-purge gas (flow rate 50 mL min^−1^). For TGA measurements, samples were placed in alumina crucibles and a TA-Q500 instrument (TA Instruments, New Castle, DE, USA) was used under the same conditions as for the DSC measurements.

A Mettler STAR^e^ TGA system (Mettler Toledo, Novate Milanese, MI, Italy) with simultaneous DSC (TGA/DSC1) was used to measure mass losses upon heating 4–6 mg samples in alumina crucibles with lids (β, N_2_-atmosphere and temperature range were the same as those for DSC above). Calibration procedure and triplicate measurements applied, as for DSC above. 

A Spectrum One FT-IR spectrophotometer (resolution 4 cm^−1^) (Perkin Elmer, Wellesley, MA, USA) equipped with a MIRacle^TM^ ATR device (Pike Technologies, Madison, WI, USA) was used for Fourier transform infrared (FT-IR) mid-IR spectroscopy (650–4000 cm^−1^) of powder samples.

Microscopic observation of sample morphologies and hot stage microscopy (HSM) were performed on samples immersed in silicone oil (heating rate β = 10 K min^−1^), under a Reichert (Arnsberg, Germany) polarized light microscope equipped with a Mettler FP82HT/FP80 system (Mettler Toledo, Novate Milanese, MI, Italy). Micrographs were recorded at various time intervals during heating with a MOTICAM 2000 video camera (Motic, Milan, Italy).

^1^H NMR spectroscopy was used to determine the host-guest ratio for the γ-CD·RSV complex. Separate solutions of the host and the guest were prepared in deuterated dimethyl sulfoxide (DMSO-d_6_). Single crystals of the inclusion complex obtained by co-precipitation were likewise dissolved in DMSO-d_6_. The proton NMR spectra of all three solutions were recorded on a Varian-Gemini 300 spectrometer (Varian, Inc., Palo Alto, CA, USA) at 298 K. Program MestreNova [36] was used to integrate non-overlapping peaks of γ-CD and RSV of known proton count in the inclusion complex spectrum. 

### 4.5. X-Ray Diffraction Methods

Powder X-ray diffraction (PXRD) patterns were collected on a Bruker D5005 powder diffractometer (Siemens, Germany) equipped with a θ–θ vertical goniometer and a Position Sensitive Detector (PSD, Braun). CuKα radiation (λ = 1.5418 Å) was employed with generator settings 40 kV and 30 mA. Patterns were recorded in the step-scan mode (step: 0.015°, counting time: 0.5 s) in the angular range 5 < 2θ° < 30 at room temperature.

For single crystal X-ray diffraction of the γ-CD inclusion complex of RSV a specimen of high crystalline quality was mounted on a nylon cryoloop with Paratone oil (Exxon Chemical Co., TX, USA) and placed on a Nonius Kappa CCD (Nonius BV, Delft, The Netherlands) diffractometer for intensity data-collection. The crystal was cooled to 173(2) K using a constant stream of nitrogen gas (flow rate 20 mL min^-1^) using a Cryostream cooler (Oxford Cryosystems, Oxford, UK). Graphite-monochromated MoKα-radiation (λ = 0.71073 Å), produced by a Nonius FR590 generator (Nonius BV, Delft, The Netherlands) operating at 53 kW and 23 mA, was employed. Data-reduction and scaling were performed using DENZO-SMN [37]. Programs in the SHELX suite [38], namely SHELXD and SHELXL, were used to solve and refine the complex structure, respectively.

## Supplementary Materials

The following are available online at https://www.mdpi.com/1420-3049/25/4/998/s1, Table S1: Solvents and volumes used in the recrystallizations of commercial RSV with thermal data by DSC of samples isolated, (Standard deviation in parentheses (*n* = 3)), Table S2: Peak assignments and integrations for host-guest stoichiometry, Figure S1: ^1^H NMR spectrum for γ-CD·RSV stoichiometry determination, Figure S2: Inclusion of an ordered guest (12-crown-4) within the channel formed by γ-CD host molecules (CSD refcode DOCYID). Figure S3: The simulated and experimental PXRD patterns of the γ-CD·RSV hydrate complex. CCDC 1974533 contains the supplementary crystallographic data for the crystal structure refinement based on the SQUEEZE routine). These data can be obtained free of charge via http://www.ccdc.cam.ac.uk/conts/retrieving.html (or from the CCDC, 12 Union Road, Cambridge CB2 1EZ, UK; Fax: +44-1223-336-033; E-mail: deposit@ccdc.cam.ac.uk).
