# Supplementary material for: Inclusion of the Phytoalexin trans-Resveratrol in Native Cyclodextrins: A Thermal, Spectroscopic, and X-Ray Structural Study
Source: Molecules. 2020 Feb 24;25(4):998. doi: 10.3390/molecules25040998 (PMC7070755; doi:10.3390/molecules25040998)
Supplement: Supplementary file 1 [file molecules-25-00998-s001.pdf]

# SUPPLEMENTARY MATERIAL, File 1

For the manuscript

**Inclusion of the Phytoalexin *trans*-Resveratrol in Native Cyclodextrins: A Thermal, Spectroscopic, and X-Ray Structural Study**

Laura Catenacci, Milena Sorrenti, Maria Cristina Bonferoni, Lee Hunt and Mino R. Caira

**Table S1.** Solvents and volumes used in the recrystallizations of commercial RSV with thermal data from DSC of samples isolated. (Standard deviation in parentheses ($n$ = 3)).

| **Solvent** | **mL per 1 g $_{RSV}$** | **$T_{onset,m}$ (°C)** | **$T_{peak,m}$ (°C)** | **$H_m$ J $g^{-1}$** |
|---|---|---|---|---|
| Water | $2.8{\cdot}10^3$ | 262(2) | 263(2) | 265(8) |
| Methanol | 17 | 262(1) | 264(1) | 237(6) |
| Methanol/water 7:3 ($v/v$) | 23 | 263(2) | 265(3) | 265(9) |
| Ethanol | 15 | 262(1) | 264(1) | 269(7) |
| Ethanol/water 1:1 ($v/v$) | 9 | 263(1) | 265(1) | 314(8) |
| Ethanol/water 4:1 ($v/v$) | 5 | 263(2) | 266(1) | 253(6) |
| Ethanol/water 7:3 ($v/v$) | 5 | 263(1) | 264(2) | 288(9) |
| Acetone | 8 | 264(1) | 265(2) | 272(3) |
| Isopropanol | 7 | 263(1) | 265(1) | 288(5) |
| n-propanol | 7 | 261(3) | 264(3) | 272(4) |
| Tetrahydrofuran | 4 | 263(2) | 265(1) | 274(5) |
| Methylene chloride | $3.7{\cdot}10^3$ | 263(1) | 265(2) | 243(5) |
| Ethyl acetate | 37 | 262(2) | 264(2) | 269(6) |

**Table S2.** Peak assignments and integrations for host-guest stoichiometry.

| **Proton (CD/G) [Total no. of protons]** | **δ (ppm)** | **Peak integrations** | **Scale factor 8 x integration value** | **Experimental peak integral /theoretical proton. No.** | **Convert to integers** |
|---|---|---|---|---|---|
| 8 x H**1** (CD) – [8] | 4.88 | 1.000* | 8 | 1 | 3 |
| 8 x O-H**6**(CD) –[8] | 4.38 | 0.95 | 8 | 1 | 3 |
| 8 x O-H**2**(CD) –[8]; 8 x O-H**3** **(CD)**–[8] | 5.62 | 1.98 | 16 | 1 | 3 |
| | | | | | |
| H**10** and H**14** (G) – [2] | 6.36 | 0.33 | 2.6 | 1.3 | 4 |
| H**3** and H**5**( G) – [2] | 7.36 | 0.35 | 2.8 | 1.3 | 4 |

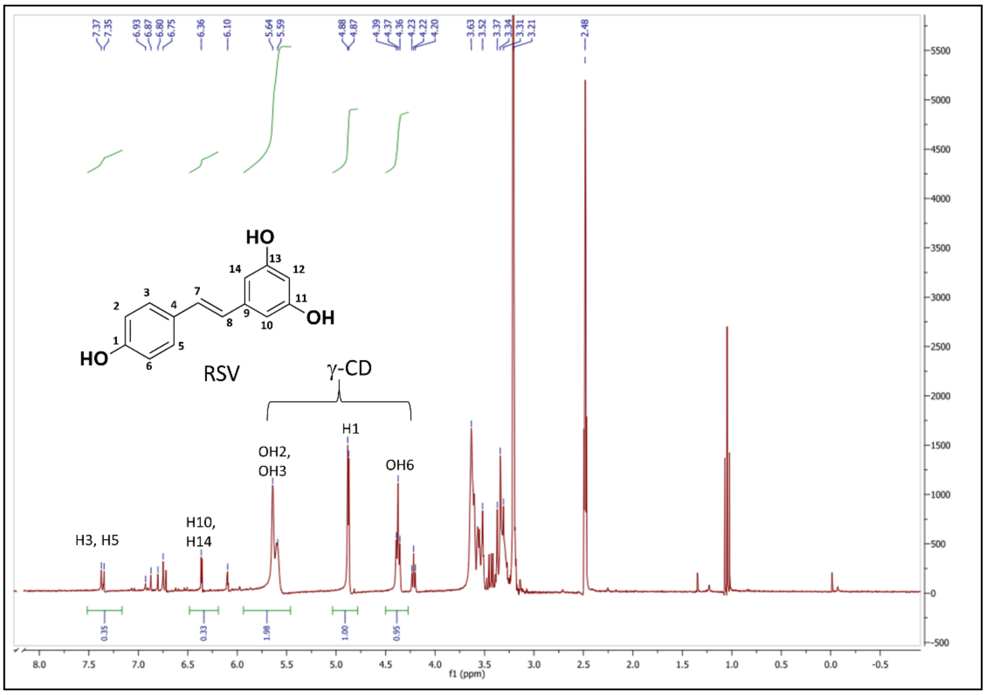


**Figure S1.** 1H NMR spectrum for γ-CD·RSV stoichiometry determination.

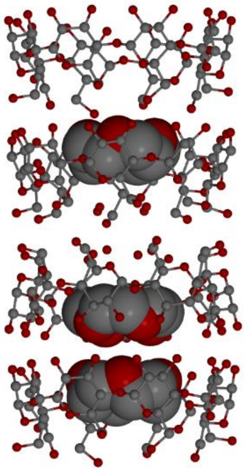

**Figure S2.** Inclusion of an ordered guest (12-crown-4) within the channel formed by γ-CD host molecules (CSD refcode DOCYID [31]). .

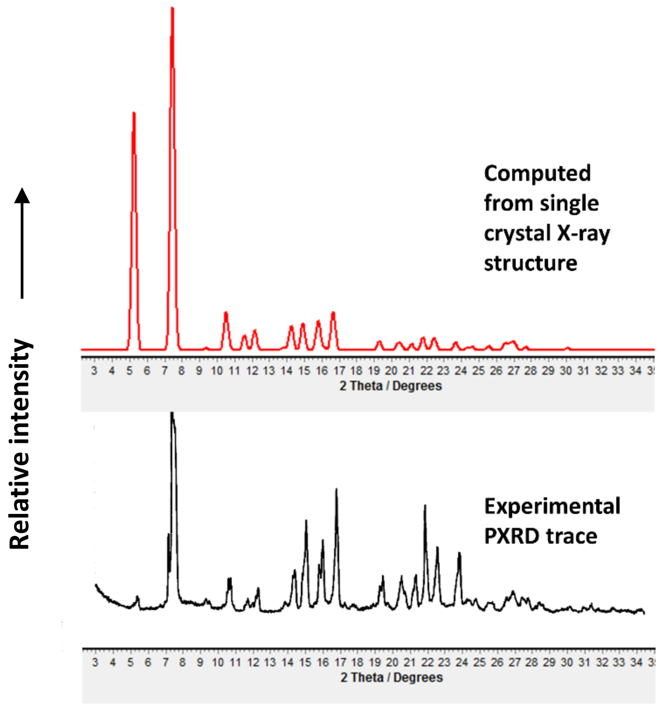


**Figure S3.** The simulated and experimental PXRD patterns of the γ-CD·RSV hydrate complex.
